# Bosnian Journal of Basic Medical Sciences becomes Biomolecules and Biomedicine

**DOI:** 10.17305/bjbms.2022.8686

**Published:** 2023-01-06

**Authors:** Muzafer Mujić, Semir Vranić, Faruk Skenderi

**Affiliations:** 1 Association of Basic Medical Sciences of Federation of Bosnia and Herzegovina, Sarajevo, Bosnia and Herzegovina;; 2 College of Medicine, QU Health, Qatar University, Doha, Qatar;; 3 Department of Pathology, UniMed Clinic, Sarajevo School of Science and Technology, Sarajevo, Bosnia and Herzegovina.

The Bosnian Journal of Basic Medical Sciences (BJBMS) was founded in 1998 in Sarajevo, Bosnia and Herzegovina, by Professor Muzafer Mujić, Professor of Physiology at the Faculty of Medicine, University of Sarajevo. Most of the scientific communication at that time, particularly in developing countries, took place through printed media with little access to a broader audience, and editorial communication took place via ground mail. Consequently, communication was much slower than today, and the journal’s readership was primarily composed of members of the local or, at best, national scientific community.

Nevertheless, the early editors of the BJBMS set high scientific standards, and this, combined with their diligence, resulted in the BJBMS being listed in PubMed/MEDLINE and Web of Science. These were the crucial initial steps in the journal’s internationalization. The journal was well received by the global scientific community, and submissions from other countries soon began to arrive.

A new era began with the widespread use of the Internet. The Internet changed almost every aspect of our lives, including our work. Scientific publishing underwent a revolution due to the vastly improved speed, efficiency, and number of communication channels. Following the trend, the BJBMS launched its first online edition, the website, in 2009. The journal’s online edition was well received by readers and increased the journal’s visibility and reputation, which was reflected in an increase in submissions from the global community. Since then, the BJBMS has experienced continuous growth in terms of quality, readership, visibility, reputation, and scientific metrics.

Today, the BJBMS is included in the most relevant scientific databases and is well-known worldwide. The journal has outgrown its national and regional boundaries given that more than 90% of the articles published in BJBMS come from the international community.

Because of aforesaid, the national designation “Bosnian” in the title of the journal does not reflect the essence and global significance of the journal. Therefore, the publisher and editors decided to change the title of the journal to more accurately reflect its international character and the content of the articles published.

From February 2023 (Volume 23, Issue 1), the title of the Bosnian Journal of Basic Medical Sciences will be changed to Biomolecules and Biomedicine. The new title reflects the increasing number of published research done on subcellular/molecular level as well as translational and clinical research contained in the term Biomedicine. Biomolecules and Biomedicine will continue to be published by the Association of Basic Medical Sciences of the Federation of Bosnia and Herzegovina.

